# Case of Vaccination Suspended by a Previous Action of Varicella

**Published:** 1803-04-01

**Authors:** N. Washbourn

**Affiliations:** Surgeon, of Marlborough, Wilts


### *Case of* VACCINATION *suspended by a previous Action of* VARICELLA; *communicated by Mr.* N. WASHBOURN, *Surgeon, of Marlborough, Wilts.*

IN the month of May, 1800, I was consulted by a friend of mine in this town respecting the Cow-Pox, and my opinion relative to its ultimate success, and prevention of the Small-Pox; I gave it, without the least hesitation, as a certain specific against the contagion of that disease; consequently, on the 15th of that month, I vaccinated two children of the family, one of whom received the infection, and went through the disease in a regular manner; but I was much surprized to find, that the younger subject, who was about a year and a half old, did not appear to be infected on the sixth day, but appeared unwell with pyrexia, which indicated the influence of some disease prevailing in the constitution.

Several in this town, and particularly one or two at the same house, were infected with the chicken-pox; no doubt, of course, could be entertained as to the probability of this complaint preventing or suspending the action of the Vaccine disease; for, on the eighth day from inoculation he sickened with the chicken-pox, and had many eruptions over different parts of his body, attended with a troublesome and teasing cough, which occasioned a complete suspension of the Vacciola until the thirteenth day after the insertion of the virus, at which time the pustules began to disappear, and the cuticle at the part incised became elevated, accompanied with inflammation: the disease now put on its regular appearance, and ended in the common way, successfully; the dark spot in the centre of the pustule did not appear until the nineteenth or twentieth day from *Inoculation*; the arm was affected with a phagedenic ulcerated appearance, either from protraction of the disease or from irritation of the child's linen, but was easily cured by the application of ung. resina flav. et hydrargy. nitrat. rub.

This case is such as to point out, clearly and evidently, the doctrine of our predecessors, of the non-existence of two diseases of a cutaneous nature prevailing at one and the same time, in the same constitution.

N. B. Since the introduction of Cow-Pox, I have had an opportunity of exposing a patient for several days, whom I inoculated with the Vaccine disease, in a house where a soldier was labouring under the confluent Small-Pox, and of of a particularly malignant nature, who with the greatest difficulty survived the disease; yet my patient resisted the action of variolous contagion, a proof of the utility of the New Inoculation, *(or Jennerian disease)* as well as a corroboration of facts already adduced, and which it is hoped will ultimately lead to a complete extirpation of the *Small-Pox.*

*Jan.* 10, 1803.

